# Self-Standing Bioinspired Polymer Films Doped with Ultrafine Silver Nanoparticles as Innovative Antimicrobial Material

**DOI:** 10.3390/ijms232415818

**Published:** 2022-12-13

**Authors:** Ekaterina A. Kukushkina, Ana Catarina Duarte, Giuseppe Tartaro, Maria Chiara Sportelli, Cinzia Di Franco, Lucía Fernández, Pilar García, Rosaria Anna Picca, Nicola Cioffi

**Affiliations:** 1 Chemistry Department, University of Bari, 70126 Bari, Italy; 2 CSGI (Center for Colloid and Surface Science), 70126 Bari, Italy; 3 IPLA—CSIC (The Dairy Research Institute of Asturias—Spanish Research Council), 33300 Villaviciosa, Spain; 4 IFN—CNR (Istituto di Fotonica e Nanotecnologie—Consiglio Nazionale delle Ricerche), 70126 Bari, Italy

**Keywords:** polymer film, polysaccharides, chitosan, silver nanoparticles, antimicrobial, synergistic, self-standing film

## Abstract

Thin self-standing films with potential antimicrobial synergistic activity have been produced by a simple green chemical synthesis with overnight thermal treatment. Their properties have been studied by scanning electron microscopy, X-ray photoelectron spectroscopy and other techniques to understand their potential range of applications. In this work, the focus was set on the development of a potential novel and effective alternative to conventional antimicrobial materials. By creating an antimicrobial polymer blend, and using it to develop and immobilize fine (~25 nm) silver nanophases, we further aimed to exploit its film-forming properties and create a solid composite material. The resulting polymer matrix showed improved water uptake percentage and better stability in the presence of water. Moreover, the antimicrobial activity of the films, which is due to both organic and inorganic components, has been evaluated by Kirby–Bauer assay against common foodborne pathogens (*Staphylococcus aureus* and *Salmonella enterica*) and resulted in a clear inhibition zone of 1.2 cm for the most complex nanocomposition. The excellent performance against bacteria of fresh and 6-month-old samples proves the prospects of this material for the development of smart and biodegradable food packaging applications.

## 1. Introduction

The rise in microbial contamination cases in recent decades, including viral pandemic and antimicrobial resistance (AMR)-driven outbreaks, calls for urgent action from the scientific community [1,2]. The application of nanotechnologies in the fields of electronics, drug delivery, and catalysis has been followed by the increased application of nanomaterials as antimicrobials [3]. Over the years, it has been shown that antimicrobial nanohybrids can be used as a powerful and safe alternative to conventional pathogen-control strategies, especially in an industrial environment [4,5,6].

Among all composites, metal-based hybrids are the most popular. In particular, silver-based nanocomposites are used in combination with peptides, polyphenols, antibiotics and other natural and/or synthetic antimicrobials [7]. To create a robust and effective system, the combination of different agents has to be beneficial for the overall antipathogen performance, e.g., showing synergistic effects [8]. In this context, nano-sized silver is well-known for its ability to release ions and reactive oxygen species (ROS), which are responsible for its bacteriostatic and bactericidal action against the majority of Gram-positive and Gram-negative bacteria, including some antibiotic-resistant strains [9,10,11]. It has been shown that, upon release, Ag^+^ can bind to several components of the cell wall, and also chemically react with the phosphate group of DNA, thus disrupting the replication process. In principle, by creating an efficient stabilizing shell around the metal core, it is possible to allow the longest possible release of active ions and continuous antimicrobial action, preventing degradation and ageing of the nanophases.

Previously, we studied the antibiofilm effectiveness of spherical colloidal nanoantimicrobials (NAMs), with an average size of around 20 nm, with fine silver nanoparticles (AgNPs) as a core and a chitosan-based polymer blend as a shell [12]. Chitosan (CS), a natural polymer with several attractive physical, chemical and biological properties, has been used for some time as a reducing and stabilizing agent in the chemical synthesis of metal nanoparticles, including silver [13]. Moreover, CS has intrinsic antimicrobial activity due to its cationic nature, and interacts with negatively charged components of the cell envelope. At the molecular level, binding of CS to the cell wall triggers secondary cellular effects which end up with a leakage of the cellular components without formation of defined pores. Another component of the polymer blend with innate antimicrobial and crosslinking properties, tannic acid (TA), is able to inhibit the adhesion of pathogen cells on different surfaces and pass through the bacterial cell wall up to the internal membrane [14].

When it comes to hybrid antimicrobials, there is no simple mechanism of action, but a complex combination of event-driven simultaneous processes. For example, taking into account two possible modes of action of the above-mentioned core–shell antimicrobial composite, the sequence of elementary events behind the antimicrobial action of CS and AgNPs may be considered as follows. First, there is a direct interaction between the CS-based polymer blend with the bacterial outer cell surface, which might be followed by its partial adsorption to the membrane, disruption of the cytoplasmic membrane, and continuous Ag ion release (indirect interaction with AgNPs); finally, a deeper penetration of the antimicrobials inside the cell may result in disruption of the whole cellular apparatus. 

In the present study, we aim to further exploit the film-forming properties of CS and its cationic polymer blend, in order to create self-standing thin polymer films. In this context, the presence of AgNPs in a homogeneous manner should provide prolonged antimicrobial activity. Keeping in mind several potential antimicrobial modes of action at the molecular level, we set out to create a robust solid system to prevent biofilm formation and kill bacterial cells upon direct interaction, avoiding resistance development [12]. Diverse key properties of the material, which could potentially be used in food packaging, were assessed through spectroscopic, microscopic, and other characterization methods. Moreover, the antimicrobial potential of the self-standing films, which is their main target feature, was evaluated against Gram-positive (*Staphylococcus aureus*) and Gram-negative (*Salmonella enterica*) bacteria.

## 2. Results and Discussion

### 2.1. Preparation of Hybrid Films

**Scheme 1:**
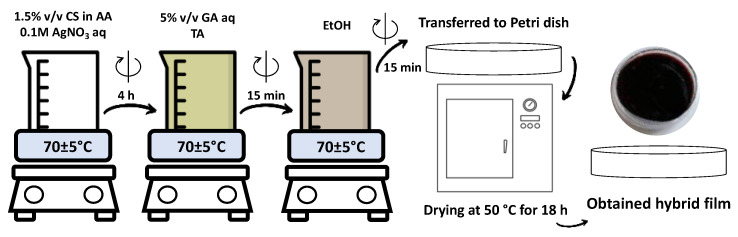
Synthetic scheme representing the development of self-standing polymer films: preparation of nanocolloids and further thermal treatment to obtain a solid-state nanocomposite.

Scheme 1 shows the main steps of the synthesis of the core–shell nanocolloids. The process has been described in detail elsewhere and is briefly described in Section 3.1 [12]. Briefly, CS and polymer blends of different compositions—with addition of crosslinkers TA and glutaraldehyde (GA)—were used as reducing and stabilizing components during the chemical synthesis of AgNPs. An aqueous system was heated in a water bath at 70 ± 5 °C, kept under constant moderate stirring and at the end of the synthesis transferred to a Petri dish with a diameter of 10 cm, placed in an oven and kept overnight at 50 °C for solvent evaporation and solidification. Self-standing thin films were taken out of the Petri dish and cut for further manipulations. 

The labeling of the different samples is presented in Table 1.

### 2.2. Microscopic Characterization of Thin Films

After drying, the films were cut into squares and analyzed visually with the naked eye and under microscopes.

#### 2.2.1. Optical Microscopy and Visual Appearance

**Figure 1:**
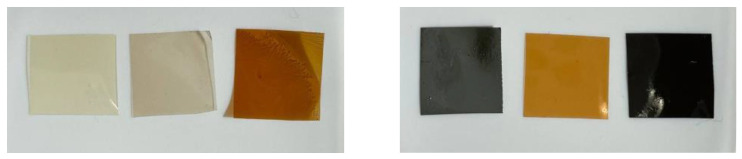
Images of self-standing polymer films of different compositions. From left to right: F1, F2, F1Ag, F2Ag, F3, F3Ag.

**Figure 2:**
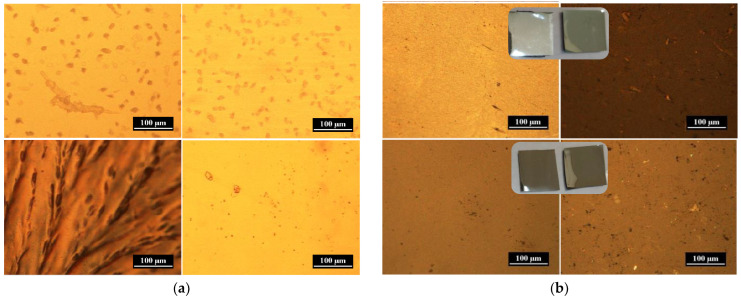
Optical microscopy (OM) images of polymer composite films: (**a**) F1 (**top**) and F1Ag (**bottom**), (**b**) F2Ag (**top**) and F3Ag (**bottom**). Surfaces of the films were studied under OM from the top (**left**) and from the bottom (**right**).

As can be seen in Figure 1, the color of the films changes depending on their composition. For instance, 20-µm-thick films appear as yellowish and translucent when pure chitosan solution was cast for drying (F1). In contrast, a greyish color is observed when TA is introduced (F2). Upon addition of silver precursor to CS (F1Ag), films display an orange color and visible crystal formation on their upper surface, an appearance that is more easily observed on the optical micrographs (Figure 2). When the CS/TA blend is combined with silver precursor (F2Ag), films appear as dark grey, with a metallic shine on their upper surface and an opaque bottom, and exhibit a higher thickness value (30 µm). Regarding films that also contain GA, F3 are orange and transparent, whereas F3Ag are dark and opaque. Both F3 and F3Ag have homogeneous surfaces and an average thickness of 25 µm. 

As can be observed in Figure 2a, sample F1Ag exhibits a drastic difference between its top and bottom surfaces, a characteristic that is not seen for sample F1, composed of only Cs. Indeed, sample F1Ag displays inhomogeneity in the crystallization pattern on the surface of the film, which might be caused by unreduced Ag^+^ ions, which are complexed with polysaccharide and forming crystals upon thermal treatment. 

Other silver-containing samples of similar compositions are shown in Figure 2b: F3Ag and F2Ag, with and without crosslinking agent, respectively. In the case of F2Ag, a shiny metal layer was noticed after thermal treatment. Therefore, for this sample there is a drastic difference between the top and bottom surfaces, which is also seen on the micrographs and photos. In contrast, both surfaces of sample F3Ag, corresponding to the most crosslinked composition, are similar and homogeneous. The distribution of metal nanophases within the films was then better investigated by electron microscopy, as discussed in the next sections. 

#### 2.2.2. Morphological Study: Scanning Electron Microscopy (SEM)

SEM analysis was performed to study the morphology and distribution of metal nanoparticles within the polymer matrix of thin films, and comparison with sample F3 without inorganic nanophases was also conducted.

**Figure 3:**
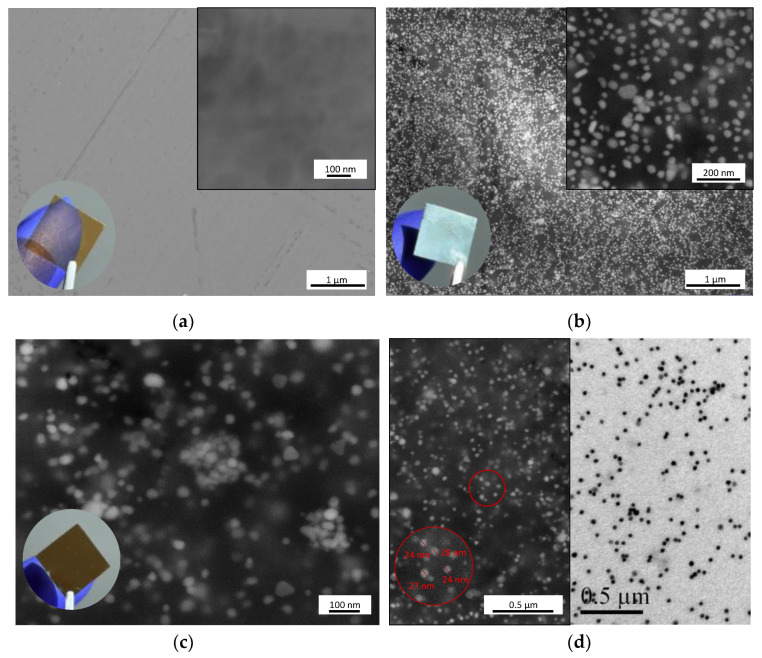
Scanning electron micrographs of polymer films: (**a**) control sample F3 without AgNPs, (**b**) F2Ag, (**c**) F3Ag and (**d**) comparison of SEM (**left**) and transmission electron microscopy (TEM, **right**) micrographs of F3Ag.

As shown in Figure 3a, the nanoparticle-free sample F3 has a uniform porous structure that can be seen at higher magnifications. In the case of sample F2Ag (Figure 3b), SEM images confirm that the metallic shine is coming from evenly distributed metal nanoparticles, which are densely packed in a layered manner. In the case of the more cross-linked, GA-containing sample F3Ag (Figure 3c), small AgNPs are incorporated within the pores of the analyzed area of the sample. The SEM and transmission electron microscopy (TEM) micrographs in Figure 3d reveal a similar distribution pattern plus the size of the solid forms (SEM, left) and colloidal mother solutions (TEM, right). To prove that NP distribution is homogeneous within the whole thickness of the film, cross-section images were taken and compared for both Ag-containing samples.

**Figure 4:**
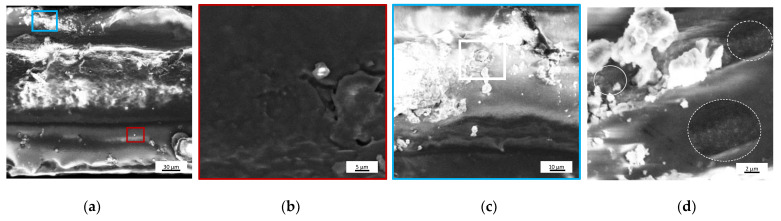
SEM images of (**a**) F2Ag sample cross-section, corresponding (**b**) higher magnification of the area highlighted in red, (**c**) higher magnification of the area highlighted in blue and (**d**) zoomed area of image (**c**).

As shown in Figure 4a, the upper part of the film had bright aggregates, which correspond to a high electron density material. Two areas of interest were identified and analyzed at higher magnifications. The first one, located deep inside the film and highlighted by a red rectangle in Figure 4a, had some silver aggregates (Figure 4b), while the second, taken in the outer layer and highlighted by a blue rectangle in Figure 4a, appears rich in bigger silver aggregates (Figure 4c). In Figure 4d, taking a closer look at the polymer matrix, it is possible to see that AgNPs appear also as small bright spots within the structure, as indicated by the white circles.

**Figure 5:**
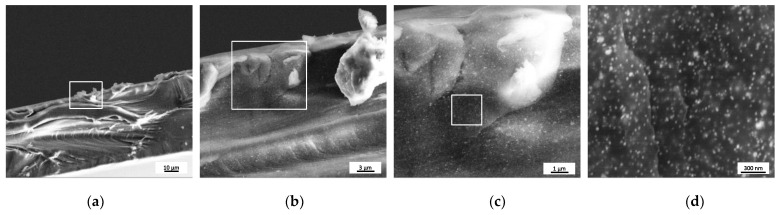
SEM images of (**a**) F3Ag sample cross-section and corresponding (from left to right) (**b**–**d**) higher magnification of the areas highlighted in white.

In contrast with the uneven distribution within the layers observed for the F2Ag sample, the polymer matrix of the GA-containing sample F3Ag presents evenly distributed nanophases without visible aggregates (Figure 5). Dispersion of small particles is detected, which is in accordance with the expectations: for colloidal solutions of the same nature, the size of the metal core was found to be around 20 nm [12]. From the images presented in Figure 5c,d, it can be gathered that AgNPs are tightly and homogeneously immobilized within the layers of the polymer blend. 

Given the notable differences in the distribution of nanosilver between the two samples shown in Figure 4 and Figure 5, it is important to understand and discuss possible explanations for the observed phenomena. In the previous study with core–shell nanocolloids, SEM analysis was performed on the colloidal solutions deposited on the solid substrate: no thin silver layer was observed on the upper part of the films for any of the tested samples [12]. These colloidal solutions, with compositions CS/TA/AgNPs and CS/GA/TA/AgNPs used as precursors for solid material, were thermally treated and resulted in the development of the self-standing films F2Ag and F3Ag, respectively. After the thermal treatment, the solid-state nanocomposite with composition F3Ag showed homogeneous distribution of fine AgNPs within the polymer, in the same manner as its precursor. In case of F2Ag, thermal treatment promoted formation of a top thin metal layer. In fact, the reductive and stabilizing powers of the polymer blends, CS/TA and highly crosslinked CS/GA/TA, are different, as was demonstrated during the wet-chemical synthesis of core–shell nanocolloids: the higher absorbance value of the nanosilver SPR maxima in the case of CS/GA/TA than in the case of CS/TA corresponds to the higher final concentration of AgNPs [12]. Moreover, it seems that there is an additional reduction occurring during the preparation of thin films. During the final thermal treatment, e.g., solvent evaporation and drying of the films, the appearance of a shiny metal layer is evidence of the incomplete reduction of residual silver precursor to AgNPs remaining after the wet-chemical synthesis. In principle, when a silver precursor is mixed with CS/TA or CS/GA/TA solution, silver ions are coordinated and form complexes with the functional groups of organic compounds, like hydroxy- and amino- groups [15]. The presence of reducing agent(s) in abundance is enough to promote the formation of Ag nuclei, whereas continuous stirring and heating during synthesis should provide the needed potential for the growth of Ag nanophases [16]. The observed differences in the final structure of the self-standing films concern the growth rate with and without the additional crosslinking agent, at the chemical synthesis stage. It seems that, when GA is present, the reduction of silver ions by the polymer blend CS/GA/TA is more complete, probably already at the chemical synthesis stage, before additional thermal treatment for creation of the final solid-state material. When CS/TA is used for stabilization and in situ reduction, additional reduction of the silver precursor is likely to occur thanks to the increase in temperature and solvent evaporation, thus creating a thin metallic layer on the surface of the film (see Figure 3b and Figure 4a). 

### 2.3. Water Uptake Measurements of Reinforced Polymer Films

**Figure 6:**
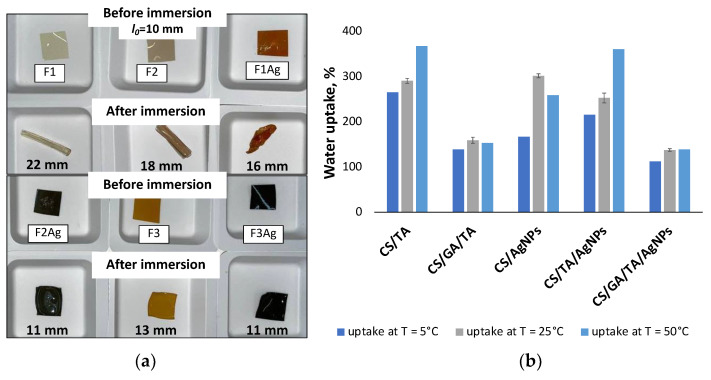
(**a**) Photos of the films before and after immersion and incubation in water, (**b**) water uptake graphs of films with different compositions under different incubation temperatures (5, 25, 50 °C).

Cs-based materials have many attractive properties, but water uptake percentage and mechanical stability in aqueous environments are not on this list. CS and many CS-based materials have poor physicochemical stability in water [17]. Figure 6 shows the improvement in the water uptake values upon addition of crosslinking agents, as well as photographs of the films before and after immersion in water, for water absorption measurements (Figure 6a). It is evident from the graphs that, when GA is present in the system (for samples F3 and F3Ag), less water is being absorbed by the composite film. For example, F2 at 25 °C has a 291% water uptake, while this value is equal to 159% when GA is present (F3) under the same incubation conditions. Upon addition of silver nanophases, the water uptake value drops independently of the presence of GA, resulting in 253% and 138% water uptake for F2Ag and F3Ag, respectively. Interestingly, all the water uptake values determined at 5, 25 and 50 °C for the sample containing a crosslinking agent (GA) decreased and leveled off at values between 110 and 155%. For all other cases, the temperature difference resulted in a higher range of % water uptake. From a practical point of view, this characteristic may represent a potential advantage in industrial applications. 

### 2.4. Thermal Stability of Thin Films: Thermogravimetric Analysis (TGA)

TGA was used to study the thermal stability of self-standing films of different compositions. The resulting TGA degradation curves and the first derivative of weight loss curves (DTG) showed changes in the decomposition patterns upon introduction of the crosslinking agents (TA and GA) and nanosilver. For all the samples, from pure CS to the most complex film, several stages of decomposition were recorded and differences in the maximal degradation temperatures were noted. 

**Figure 7:**
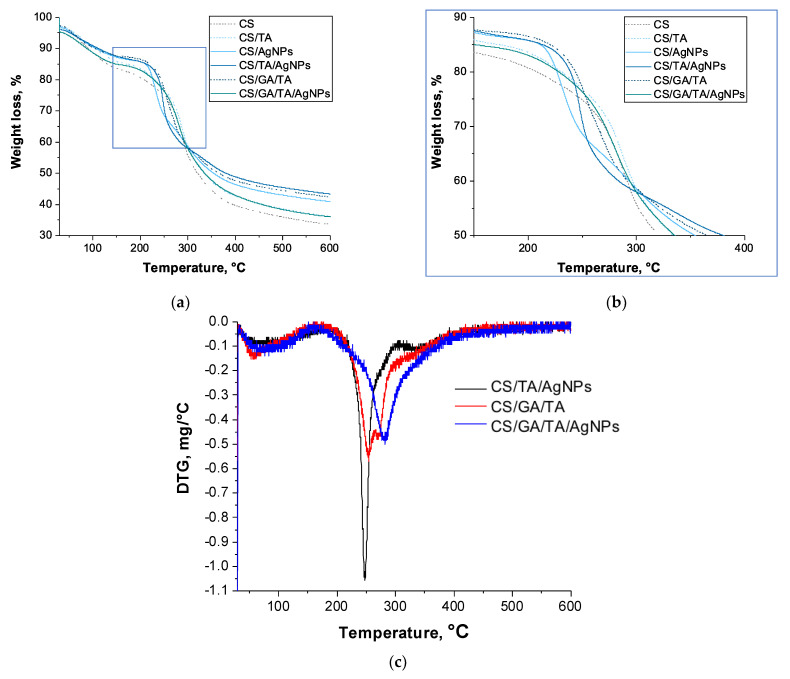
(**a**) Thermogravimetric analysis (TGA) curves, (**b**) zoom of the area highlighted in blue in the former panel and (**c**) first derivative of weight loss curves (DTG) of composite films.

Table S1 shows the peak decomposition temperatures, weight loss percentage and corresponding masses. All the changes are most probably associated with the chemical and structural changes taking place within the polymer matrix, when additional components are added to bare CS film. These changes have a complex nature, as can be seen from the different degradation patterns of the films shown in Figure 7.

F1, F2, F1Ag, F2Ag and F3 showed threefold thermal degradation, while F3Ag underwent two clear degradation events within the same temperature range (Figure 7c). For all of the samples, the initial weight loss was recorded at ~50–90 °C and can be attributed to the vaporization of the residual moisture from the porous films (mass loss from 14.1% for CS films to 12.3% for CS/GA/TA/AgNPs). The second maximum thermal degradation temperature lay in the range from 210 to 255 °C and, according to literature [18], it might be attributed to the initial decomposition of the polymer matrix and the final removal of volatile products. 

Upon addition of TA, silver precursor, and the crosslinking agent GA, the maximum degradation temperature gradually increases at this step in a manner to the degree of crosslinking. One of the samples with the highest crosslinking degree, F3, showed two peaks in the same temperature range, at 253 °C (13.8% weight loss) and 270 °C (14.5% weight loss), as shown on the DTG graph in Figure 7c. The reported degradation temperature values of neat chitosan have a broad range, since they are highly dependent on the crystallinity, deacetylation degree and molecular weight of the chitosan precursor, as well as the selection of the solvent and film preparation procedure [19]. In general, weight losses in this temperature range (250–280 °C) could be associated with the initial degradation of the polysaccharide polymer structure. For the complex compositions, the major weight loss observed in this range could also be attributed to the breakage of the CS-TA and CS-GA-TA bonds. For F3Ag, upon introduction of AgNPs into the CS/GA/TA system, only one sharp peak appears at 282 °C, with a maximum simultaneous weight loss of 39.3%. In the literature, for the same thermal range, partial decomposition of TA and thermal degradation of amine units of CS have been reported at ~280–310 °C and 255–320 °C, respectively [18,20]. At the same time, the inhomogeneous structure of F2Ag has two distinct degradation T peaks associated with polymer structure decomposition at 248 °C (major weight loss 28.3%) and at 335 (9.1%). The second weight loss happened in the broad temperature range from 310 to 380 °C, and was probably associated with further polymer matrix decomposition. This might hint at the importance of crosslinking homogeneity within the film structure. Moreover, it is likely that even the dispersion of silver and the presence of GA are playing important roles on the structural properties of the matrix.

### 2.5. Spectroscopic Characterization of the Films

#### 2.5.1. Fourier Transform Infrared (FTIR) Spectroscopy

FTIR spectroscopy was used to characterize the effect of addition of crosslinking agents (TA and GA) and inorganic phases (AgNPs) to chitosan films.

**Figure 8:**
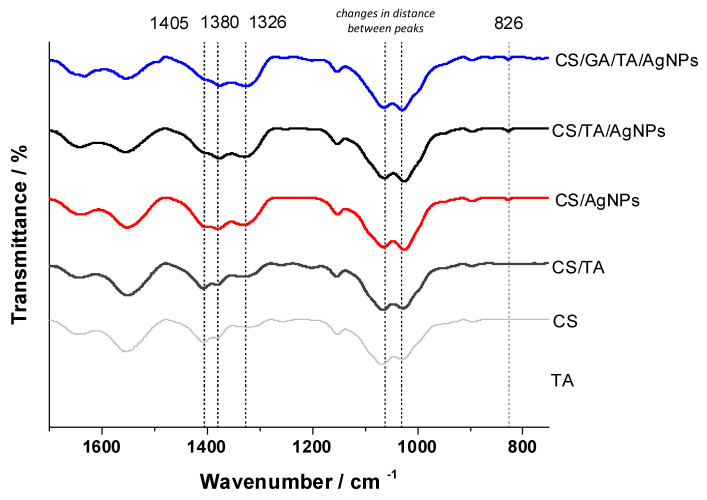
FTIR spectra of composite films, fingerprint region: control of tannic acid (TA), F1 (CS), F2 (CS/TA), F1Ag (CS/Ag), F2Ag (CS/TA/AgNPs), F3 (CS/GA/TA/AgNPs).

The FTIR fingerprint region spectra are shown in Figure 8 for all the sample sets. Broad bands at 1640, 1542 and 1024 cm^−1^ can be attributed to the signals coming from the N- and O-rich groups, in particular: C=O stretching of the amide I, NH-bending vibrations of amide II and C-O stretching, respectively [21]. Band positions and attributions are listed in Table 2.

Crosslinking with reagents like TA and GA leads to drastic changes in spectra due to the electrostatic and chemical interactions between functional groups. Upon crosslinking with TA, there were noticeable relative intensity changes in the peaks observed at 1405 and 1380 cm^−1^, which would be associated with the CH_3_ deformation mode, as well as broadening of the C-N stretching band (1326 cm^−1^). The absence of a band at around 1450 cm^−1^ on the spectra of CS combined with TA can be attributed to the tight interaction and possible complexation of the carboxyl group of TA with the CS molecule. Upon the addition of silver (F1Ag and F2Ag), there is a decrease in the intensity of the abovementioned peaks, highlighting the active interaction of this metal with the functional groups of CS and TA. Furthermore, for the most complex sample, F3Ag, we saw an attenuation of the relative intensities of the same peaks in comparison with simpler compositions. The most concerning aspects were the changes in the peak separation distances associated with C-O-C bonding vibrations, which are due to electrostatic interactions and hydrogen bonding. The peak at 826 cm^−1^, according to the literature, represents the interaction of oxygen with metal, e.g., Ag– O, in the silver-containing films [22,23]. 

#### 2.5.2. Surface Analysis by X-ray Photoelectron Spectroscopy (XPS)

X-ray photoelectron analysis (XPS) was used to probe the chemical composition of the surface of the self-standing films. The main elements found in the analyzed films were carbon (C), oxygen (O), nitrogen (N) and, in the samples of interest, silver (Ag). Additional elements typically present in CS powder were also detected in trace amounts (e.g., Ca, Si, I). 

The results of this analysis are presented in Table 3, showing drastic differences in silver abundance in the outer few nanometers of the surface of CS/TA/AgNPs and CS/GA/TA/AgNPs films.

**Figure 9:**
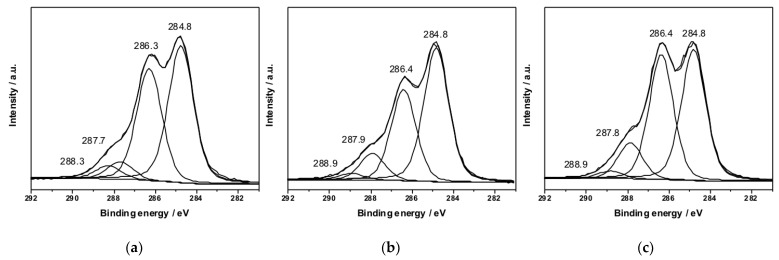
C1s XP deconvoluted spectra relevant to: (**a**) F3 (CS/GA/TA), (**b**) F2Ag (CS/TA/AgNPs), and (**c**) F3Ag (CS/GA/TA/AgNPs) films.

Such evidence is not surprising, considering the different distribution of Ag nanophases in the two samples, as observed by SEM analysis (Figure 4 and Figure 5). In fact, the AgNPs present in F2Ag samples appear mainly on the upper surface, connecting to each other and forming a shiny metal-like layer. On the other hand, the addition of another crosslinking agent, such as GA, results in more separated inorganic NPs that appear tridimensionally distributed in the polymeric matrix. C1s XP spectra of F3, F2Ag, and F3Ag are shown in Figure 9. No qualitative differences arose along the series, always revealing the presence of four peaks for all the analyzed samples. Some minimal differences were found in quantitative aspects. Table 4 summarizes the peak position and relative abundance of the C1s peak components for the three films. All the assignments are in agreement with previous literature [24,25]. The component at 284.8 ± 0.2 eV is always the most abundant, due to CS crosslinking. The inclusion of AgNPs does not particularly affect the carbon chemical environment. The sample showing the best conversion of the silver precursor into finely and tridimensionally dispersed AgNPs (F3Ag) just shows a slightly higher cumulative abundance of oxidized carbon species. For F3Ag, the sum of the three non-aliphatic peaks is indeed equal to 53%, while it is ≤50% for the other samples of Table 4. 

**Figure 10:**
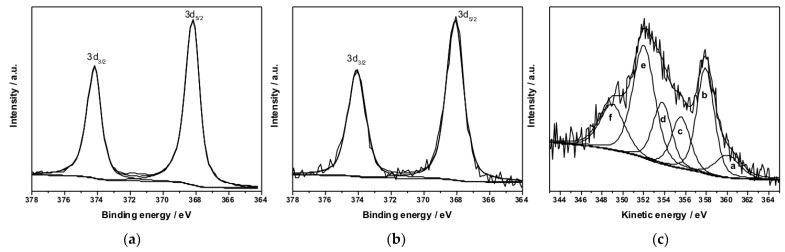
Ag3d XP deconvoluted spectra relevant to: (**a**) F2Ag and (**b**) F3Ag. (**c**) AgM_4,5_N_45_N_45_ region relevant to F2Ag films.

To identify the chemical state of silver, Ag3d and modified Auger parameter were used. Ag3d transition is poorly sensitive to the chemical shift; therefore, a careful analysis of the additional AgM_4,5_N_45_N_45_ Auger signal was needed to estimate silver speciation at the surface. In fact, in the case of F2Ag (Figure 10a) and F3Ag (Figure 10b), Ag3d_3/2_ components appear at the following BE values: 374.2 ± 0.2 eV, and 374.1 ± 0.2 eV, respectively. As reported in Figure 10c, it was possible to acquire Auger signal for F2Ag samples. 

Ag M_4,5_N_45_N_45_ was fitted with six peaks (a–f), according to previous literature [26,27]. The modified Auger parameter (α′) was calculated as the sum of BE (Ag 3d_5/2_) and the KE of the sharpest peak (b) in Figure 10c (357.9 ± 0.2 eV) and was found to be 726.1 ± 0.3 eV. Moreover, the independent parameter *R*, namely the d/e peak area ratio, is equal to 0.46 ± 0.05. Both calculated α′ and *R* values, according to the literature, correspond to Ag(0) [26,28], indicating a successful reduction of the Ag^+^ precursor. 

### 2.6. Antimicrobial Tests

Since the developed thin self-standing films are potentially applicable as a food packaging material, antimicrobial experiments against common food industry-related pathogens were performed. 

#### 2.6.1. Optimization of Test Conditions

Before performed the antimicrobial assays, the compatibility of the growth medium with the developed materials was tested.

Depending on the bacterial strain and the experiment to be performed, the composition of the medium and the percentage of agar was shown to be important. For the selected bacteria, *S. enterica* (SE) and *S. aureus* (STAPH) and for the Kirby–Bauer disk diffusion method, several requirements were taken into consideration, namely good growth of the chosen bacteria, proper agar concentration to allow homogeneous and good diffusion of the antimicrobial agent and, most importantly, absence of side reactions between the medium and the antimicrobial components. 

**Figure 11:**
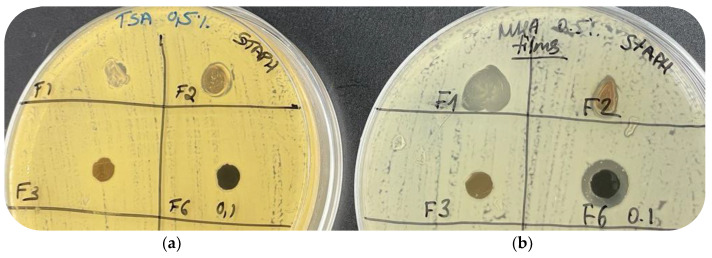
Results of the optimization of the disk diffusion test conditions on: (**a**) tryptic soy agar 0.5% (TSA) and (**b**) Mueller–Hinton agar 0.5% (MHA).

At first, tryptic soy broth (TSB) or tryptic soy agar (TSA) were chosen for antimicrobial tests. No inhibition zone was observed for any of the samples, despite the expectations and the reported results in the literature (Figure 11a). Additional experiments in liquid medium and CS solution were carried out, showing time-dependent appearance of turbidity and precipitation (Figure S1). Indeed, it is possible that some TSB components are able to chemically react with the polymer blend. For example, it is well-known that casein, one of the main components of TSB, can form complexes with chitosan [29,30]. Thus, this side reaction might be responsible for the absence of visual antibacterial activity.

To minimize the influence of undesired chemical reaction(s), screening for a suitable alternative took place and Mueller–Hinton broth (MHB) or Mueller–Hinton agar (MHA) were selected as an alternative option. Replacement of TSA with MHA gave a positive result and clear inhibition zones were detected for silver-containing films (Figure 11b).

#### 2.6.2. Disk Diffusion Method to Test Developed Films against Foodborne Pathogens

The disk diffusion method was chosen to assess the antimicrobial activity of the developed thin films, since it is one of the most commonly used to test materials. 

The biocidal activity of films with different compositions was evaluated qualitatively against common foodborne pathogens *S. enterica* and *S. aureus*, which are responsible for frequent cases of food contamination at the industrial level worldwide. These pathogens are easy to grow overnight at 37 °C even under static conditions. After 16 h of bacterial growth, the diameter and visual appearance of the inhibition zones were examined for disks taken from films with different compositions as an indication of bacterial susceptibility to a given film. Although all the different components used for film synthesis may potentially exhibit antimicrobial properties [7,31,32], we focused our attention on the role exerted by silver in the most complex and stable (towards water exposure) films.

**Figure 12:**
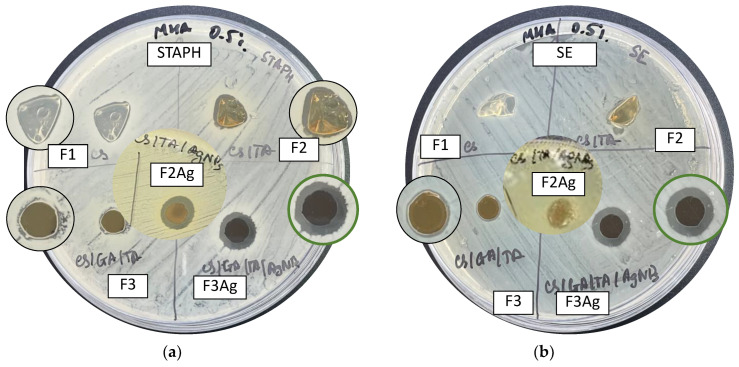
Results of the disk diffusion tests for hybrid films (F1 (CS), F2 (CS/TA), F2Ag (CS/TA/AgNPs), F3 (CS/GA/TA), F3Ag (CS/GA/TA/AgNPs) against (**a**) *S. aureus* and (**b**) *S. enterica*.

Figure 12 shows agar plates inoculated with *S. aureus* (a) or *S. enterica* (b) with disks after overnight incubation at 37 °C. There were several notable outcomes. First, it is evident that pure CS (F1) and CS/TA (F2) films exhibit changes in their shape and state during the incubation time, due to exposure to a humid environment, in accordance with the water uptake measurements results and discussions of the crosslinking influence on the mechanical performance of the films. A weak halo and small inhibition zone appear around disks corresponding to samples that did not contain AgNPs. On the one hand, the appearance of a clear inhibition zone for both Gram-positive and Gram-negative bacteria was observed for samples F2Ag and F3Ag. In both cases, the inhibition diameter was equal to 1.2 ± 0.1 cm (d_disk_ = 0.6 cm), as reported in Table 5. On the other hand, after incubation, sample F2Ag showed changes in color—this indicates ongoing chemical transformations when films are brought into contact with MHA and/or bacteria. In the case of the most complex composition, no mechanical changes were observed (expansion in size or changes in conformation).

Summarizing the outcomes of the disk diffusion assay, it is safe to say that we observe synergistic action of the nanohybrids. At the molecular level, we expect to see the evidence of the cationic nature of the polymer blend and ion release and further diffusion of the silver species into the agar. For both *S. aureus* and *S. enterica*, we observed a small and uneven inhibition zone in the case of sample F3, i.e., the silver-free sample with the highest crosslinking degree and complexity (Figure 12). Thus, the clear inhibition zone displayed by disks containing F3Ag is most probably the outcome of both the cationic effect of the polymer and the silver species. It might be that the polymer, when in direct contact with bacteria, disrupts the permeability of the cell envelope, thereby facilitating the higher influx of silver ions into the cell, which ultimately leads to a more pronounced bactericidal effect. 

Moreover, in order to further evaluate the beneficial effect of inorganic nanophase, silver ion release tests were made for the most complex and biologically effective silver-containing sample (F3Ag) and its metal-free analogue (F3). On the Figure S2 kinetics release of sample CS/GA/TA/AgNPs (0.1 M) is shown, resulting in 6, 13 and 22 ppb after 6 h, 12 h and 36 h of sampling, respectively. The polymer blend with high crosslinking degree creates a tight protective organic shell, which allow release of the ions in controlled and prolonged way. In this way, we can relate clear inhibition zones observed by disk diffusion assay for the F3Ag sample with the efficient and long-lasting release of ions.

**Figure 13:**
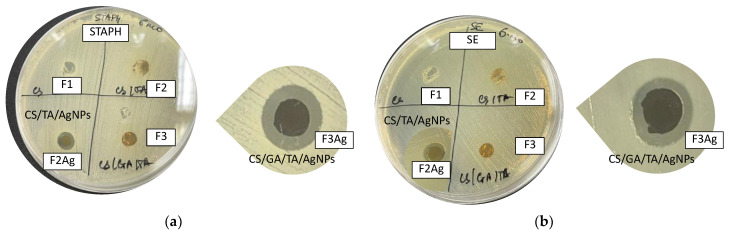
Results of disk diffusion tests for hybrid 6-month-aged films (F1 (CS), F2 (CS/TA), F2Ag (CS/TA/AgNPs), F3 (CS/GA/TA), F3Ag (CS/GA/TA/AgNPs) against (**a**) *S. aureus* and (**b**) *S. enterica*.

To assess the potential of these thin films in industrial applications, it is important to address the existence of some desirable long-lasting properties, especially antimicrobial efficiency after long-term storage. To do so, the very same set of disk diffusion experiments was carried out with 6-month-old films. However, as is shown in Figure 13, only silver-containing samples kept their bactericidal action after storage. Indeed, for silver-containing samples, the diameter of the inhibition zones was almost the same as for the freshly prepared films, e.g., 1.2 ± 0.1 cm in the case of *S. aureus* (Figure 13a, Table 5) and 1.1 ± 0.1 cm for *S. enterica* (Figure 13b, Table 5). This is reasonably due to the better stabilizing effect offered by the specific composition of these samples.

## 3. Materials and Methods

### 3.1. Materials and Preparation of Hybrid Films

Thin polymer films were reproducibly prepared using solvent evaporation method. Starting polymer CS solution (5 mL of 1.5% *w*/*v* using 1% aqueous acetic acid as solvent) was combined with aqueous silver precursor (1 mL of 0.1 M AgNO_3_) and left under constant moderate stirring and heating (60 ± 5 °C) for 4 h in the dark conditions. By the end of the reaction time, the reaction solution turned into yellow, signaling on AgNPs formation. This solution was used for the creation of F1Ag film. To create a more complex system, at the end of the AgNPs synthesis, TA only (0.008 mg) or GA (0.15 mL of 5% *w*/*w* aqueous) and TA were introduced with a timestep of 15 min and further used for a thermal overnight treatment to develop F2Ag and F3Ag, respectively. Color changes from yellow to dark grey (F2Ag), and dark brown (F3Ag) were used as indication of complete reaction. Silver free solutions were made to develop F1, F2 and F3 by introducing 1 mL of H_2_O (Milli-Q) instead of silver precursor. Solutions were transferred to Petri dishes (10 cm) and treated at 50 °C overnight to obtain the final films. 

Chitosan (Cs) (medium molecular weight and 75–85% deacetylation degree), and purified glutaraldehyde solution (grade I) of 25% in H_2_O were used. All the chemicals used for synthesis and sample preparation were of analytical grade and purchased from Sigma-Aldrich, Germany. The thickness of the freshly prepared films was determined by manual caliper. All the measurements were performed in triplicates.

### 3.2. Morphology and Particle Distribution

Optical micrographs were taken with Nikon Eclipse (ME600, Nikon, Tokyo, Japan) under different magnifications showing the difference under the same parameters, e.g., light brightness. The images were taken from both sides of the film after drying: the side which was exposed to air and the side which was attached to the bottom of the Petri dish

The surface and cross-sectional morphologies of thin self-standing polymer films and AgNPs distribution within the matrix were investigated with a scanning electron microscope (SEM), Zeiss (Field Emission Σigma), Germany, at accelerating voltages of 5 (control CS/GA/TA sample) and 10 kV (all other silver-containing samples). For surface morphology studies, films were directly placed in the chamber for a measurement without any additional treatments. For cross-section experiments, pieces of film were placed in liquid nitrogen and further broken into smaller pieces.

Transmission electron microscopy (TEM) was used to double-check the generation of the AgNPs in the chitosan-based film after their in situ synthesis. JEOL JEM −1011 microscope was used at an acceleration voltage of 100 kV. TEM samples were prepared by placing several microliters of diluted solutions on 300 mesh copper grids, dried and transferred to the chamber for analysis. 

### 3.3. Water Uptake Measurements

Water uptake percentage was studied for the films of different compositions. Freshly prepared samples of approximately 10 × 10 mm were cut and the initial weight was noted. Then, samples were placed in vials with 10 mL of Milli-Q water at temperatures: 5, 25, 50 °C and left for 2 h with the temperature control. Afterwards, swollen samples were removed from the water, and the excess water was removed from the surface by using filter paper, prior to measuring the final weight. To evaluate the water uptake percentage, the following formula (1) was used:

(1) $$\%\;\mathrm{water}\;\mathrm{uptake}=\left[\frac{W_{s}-W_{i}}{W_{i}}\right]\times 100$$

where *W_s_* and *W_i_* are the weights of the swollen and initial films, respectively. Measurements were performed in triplicate.

### 3.4. Thermogravimetric Analysis 

TGA of complex polymer films was performed using Perkin Elmer Pyris TGA. Samples of 5–10 mg were heated from 30 (held for 5 min) to 600 °C (held for 5 min) in 10 °C/min steps under constant nitrogen flow (20 mL/min). The TGA data were used to plot TGA (weight loss vs. temperature) and the first derivative of the weight loss DTG curves.

### 3.5. Fourier Transform Infrared Spectroscopy

The Fourier transform infrared (FTIR) spectra of the films were recorded at room temperature in a Perkin Elmer Spectrum Two FTIR spectrophotometer with a DTGS detector in transmittance mode (scan number 32, resolution 2 cm^−1^, range 4000–400 cm^−1^) and processed using the Spectrum software. Attenuated Total Reflection (ATR) accessory equipped with diamond prism was used, placing small pieces of the films directly on the crystal for their analysis. 

### 3.6. X-ray Photoelectron Spectroscopy

X-ray photoelectron spectroscopy (XPS) analysis was performed to determine chemical speciation of thin films surfaces. For this, PHI 5000 Versa Probe II Scanning XPS Microprobe spectrometer (ULVAC-PHI Inc., Kanagawa, Japan) was used. The measurements were performed with a monochromatized Al Kα source (X-ray spot 200 µm), at a power of 50.3 W. Both wide and detailed spectra were acquired in fixed analyzer transmission (FAT) mode with a pass energy of 117.40 and 58.70 eV, respectively. An electron gun was used for charge compensation (1.0 V 20.0 µA). Calibration of the binding energy (BE) scale was performed by fixing the aliphatic component of the C 1s signal (BE = 284.8 ± 0.1 eV) as reference. Samples composition was determined by means of atomic %. Data treatment was done by using the MultiPak software v. 9.9.0.8 (Kanagawa, Japan). Errors were calculated on the basis of 3 different measurement points.

### 3.7. Antimicrobial Activity

**Figure 14:**
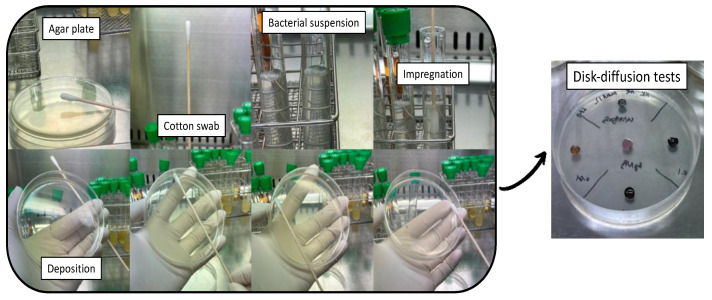
Preparation of the samples for the disk diffusion assay.

To test antimicrobial susceptibility of the developed composite films, Gram-positive *S. aureus* and Gram-negative *S. enterica* were chosen. The activity was evaluated using the Kirby–Bauer disk diffusion method. Briefly, a bacterial suspension from an overnight culture, grown in TSB at 37 °C, was diluted in Phosphate Buffer Saline (PBS) to reach a final concentration of ~10^7^ CFU/mL or OD (600 nm) = 0.1. Then, the freshly prepared bacterial suspension was spread on MH plates containing 0.5% agar as shown in Figure 14.

Small disks of composite films with a diameter of 0.6 cm were accurately placed onto the inoculated agar, and then incubated at 37 °C for 16–18 h. The resulting inhibition zones/halos around the disks were measured. Plates were left for an additional check for another 16 h.

## 4. Conclusions

In this work, thin polymer films were developed using a fast and versatile synthesis method, including thermal treatment, and characterized from several points of view. The composition had various grades of complexity: F1Ag (CS/AgNPs), F2Ag (CS/TA/AgNPs), F3Ag (CS/GA/TA/AgNPs) and their silver-free analogs. The distribution of nanosilver resulting in interesting outcomes. First, it was found that the thin metal layer of the sample F2Ag corresponded to a tight network of AgNPs, while the cross-section studies revealed uneven dispersion of the inorganic phase within the layers of polymer matrix. In the case of F3Ag, excellent homogeneous distribution with 6-month stability of the inorganic phases within the matrix was found by both surface scanning and cross-sectional analysis. Both silver containing materials (F2Ag and F3Ag) could be used as antimicrobial self-standing films, although they showed different stability towards water exposure. On a molecular level, both spectroscopic characterization, which revealed changes in bond formation and interaction of various functionalities of the components and also the differences in surface composition, and microbiological experiment outcomes demonstrated the potential of the nanohybrids to be used in the food sector. By XPS, the chemical state of silver, Ag(0), was found by attributing BE of the Ag3d components and by calculating the modified Auger parameter (α′). Antimicrobial tests against common food contaminants, *S. enterica* and *S. aureus*, proved the antipathogen potential of this material: the appearance of clear inhibition zones for both Gram-negative and Gram-positive strains showed good diffusion of the antimicrobial agent and the corresponding toxicity of the films for the selected pathogens. The most pronounced effect was observed for theF2Ag and F3Ag samples, due to both the cationic nature of the polymer blend and the controlled ion release from the evenly distributed AgNPs. Water uptake measurements showed lower swelling extent at different temperatures (5, 25 and 50 °C) for all cross-linked, GA-containing samples. The resulting limited changes in physical appearance suggest that F3Ag films could be better suited to industrial use.

Polymer films are prone to ageing, and NPs could undergo phase separation [33]. Moreover, the composite swelling is anticipated to be strictly dependent on the lipophilic character of the food matrix. Investigations are therefore planned on a time scale longer than 6 months, and towards the storage of different food-mimicking media.

In conclusion, a novel organic/inorganic solid hybrid material, specifically a highly crosslinked polymer film with tightly immobilized nanophases, possesses strong bactericidal properties. Due to the synergistic mechanism of inhibition, the self-standing films developed in the present study hold promising potential to prevent the development of antimicrobial resistance. Taking all of this into account, we say that this composite film has an excellent set of properties which are needed for industrial applications, in particular as part of a smart eco-friendly food packaging material. Work is in progress to combine the F3Ag material with biodegradable polymers, such as poly lactic acid, in a layered structure, thus minimizing the possible drawbacks related to the film swelling, and also reducing the cost of the final package.

## Figures and Tables

**Table 1:** Sample labeling of thin polymer films.

Composition of Thin Films	Sample Label
CS	F1
CS/TA	F2
CS/AgNPs	F1Ag
CS/TA/AgNPs	F2Ag
CS/GA/TA	F3
CS/GA/TA/AgNPs	F3Ag

**Table 2:** FTIR bands of composite polymer films.

Functional Group	Wavenumber (cm ^−1^)	Description
C=O	1632	Interactions of the amide group I (CONH_2_) with the functional groups of TA upon crosslinking cause pronounced C=O stretching
NH_3_^+^ symmetrical deformation	~1540	Interactions of CS with TA in acidic conditions cause ionization of the primary amino group II in the presence of carboxylic group
CH_3_ symmetrical deformation mode	1405	Changes of the relative peak intensities due to the interaction with TA
	1380	
C-N stretching	1326	Strong signal from TA, blue shift and decrease in the intensity due to the interaction with CS
C-O vibration	1200	Disappearance of the band upon addition of AgNPs
OH bending vibration	~1145	Due to the structure of the saccharide
C-O-C bonding vibrations	1100–1000	Peak separation changes due to the crosslinking and functional groups interaction
AgNPs	826	Interaction of AgNPs with O- and N-rich functional groups

**Table 3:** Typical surface composition for F3, F2Ag, and F3Ag films. Error refers to standard deviation calculated in at least three replicates. When this parameter was lower than the error associated with a single elemental quantification, the latter was taken as uncertainty of measure.

Sample Composition	F3 (CS/GA/TA)	F2Ag (CS/TA/AgNPs)	F3Ag (CS/GA/TA/AgNPs)
Element	Atomic %		
C	74 ± 3	65.2 ± 0.5	69 ± 2
O	22 ± 2	25.6 ± 0.5	26.3 ± 1.5
N	3.6 ± 0.5	4.6 ± 0.5	4.5 ± 0.6
Ag	/	2.9 ± 0.2	0.2 ± 0.2
Si	0.4 ± 0.5	1.1 ± 0.5	<0.5
Ca	<0.5	0.6 ± 0.5	<0.5
I	<0.2	<0.2	<0.2

**Table 4:** C1s components identified for F3, F2Ag, and F3Ag samples. The maximum error on the peak position is ±0.2 eV.

Sample	Component	Assignment	Relative Abundance%
F3	284.8	C-C, C-H	50 ± 3
	286.3	C-O, C-N	39 ± 4
	287.7	O-C-O, C=O	6.2 ± 0.9
	288.3	COOH, N-C=O	4.8 ± 0.7
F2Ag	284.8	C-C, C-H	52 ± 5
	286.4	C-O, C-N	36 ± 4
	287.9	O-C-O, C=O	10 ± 2
	288.9	COOH, N-C=O	2 ± 1
F3Ag	284.8	C-C, C-H	47 ± 5
	286.4	C-O, C-N	42 ± 4
	287.8	O-C-O, C=O	9 ± 2
	288.9	COOH, N-C=O	2 ± 1

**Table 5:** Summary of zone of inhibition diameters of thin fresh and aged polymer films after disk diffusion assay. The initial sample diameter is 0.6 cm.

Sample	Composition	Zone of Inhibition of Fresh Sample, cm		Zone of Inhibition of Aged Sample, cm	
		*S. aureus*	*S. enterica*	*S. aureus*	*S. enterica*
F1	CS	0.7 ± 0.1	0.6 ± 0.1	0.6 ± 0.1	0.6 ± 0.1
F2	CS/TA	0.7 ± 0.1	0.6 ± 0.1	0.6 ± 0.1	0.6 ± 0.1
F3	CS/GA/TA	0.7 ± 0.1	0.7 ± 0.1	0.6 ± 0.1	0.6 ± 0.1
F2Ag	CS/TA/AgNPs	1.2 ± 0.1	1.1 ± 0.1	1.0 ± 0.1	1.1 ± 0.1
F3Ag	CS/GA/TA/AgNPs	1.2 ± 0.1	1.2 ± 0.1	1.2 ± 0.1	1.1 ± 0.1

## Data Availability

The data presented in this study are available on request from the first and the corresponding authors.

## Supplementary Materials

The following supporting information can be downloaded at: https://www.mdpi.com/article/10.3390/ijms232415818/s1, Table S1: The summary of thermogravimetric analysis: the peak decomposition temperatures, weight loss percentage and corresponding masses for the thin films of different compositions; Figure S1: Compatibility tests of hybrid nanocomposites and culture medium, tryptic soy broth (TSB).
