# Supplementary material for: Self-Standing Bioinspired Polymer Films Doped with Ultrafine Silver Nanoparticles as Innovative Antimicrobial Material
Source: Int J Mol Sci. 2022 Dec 13;23(24):15818. doi: 10.3390/ijms232415818 (PMC9779273; doi:10.3390/ijms232415818)
Supplement: Supplementary file 1 [file ijms-23-15818-s001.zip › ijms-2036849-supplementary.pdf]

International Journal of *Molecular Sciences*

MDPI

Article

Self-Standing Bioinspired Polymer Films Doped with Ultrafine Silver Nanoparticles as Innovative Antimicrobial Material

Ekaterina A. Kukushkina [1,2], Ana Catarina Duarte [3], Giuseppe Tartaro [1,2], Maria Chiara Sportelli [1], Cinzia Di Franco [4], Lucía Fernández [4], Pilar García [4], Rosaria Anna Picca [1,2] and Nicola Cioffi [1,2,*]

1 Chemistry Department, University of Bari, 70126 Bari, Italy
2 CSGI (Center for Colloid and Surface Science), 70126 Bari, Italy
3 IPLA—CSIC (The Dairy Research Institute of Asturias—Spanish Research Council), 33300 Villaviciosa, Spain
4 IFN—CNR (Istituto di Fotonica e Nanotecnologie—Consiglio Nazionale delle Ricerche), 70126 Bari, Italy
* Correspondence: nicola.cioffi@uniba.it

Supplementary Information

Table S1. The summary of thermogravimetric analysis: the peak decomposition temperatures, weight loss percentage and corresponding masses for the thin films of different compositions.

Sample name	M_0, mg	T_1 max, °C	Δm_1, mg	T_2 max, °C	Δm_2, mg	T_3 max, °C	Δm_3, mg	M, mg	Total % weight loss, %
F1	5.601	83.5	0.958 (14.1%)	210	0.353 (6.3%)	285.3 292.2	2.032 (36.2%)	2.258	56.6
F2	5.163	88.7	0.749 (14.5%)	228.5	0.314 (6.1%)	286.8	1.764 (34.2%)	2.336	54.8
F1Ag	6.488	53.2	0.875 (13.5%)	233.5	1.139 (17.6%)	296.3	1.177 (18.1%)	3.297	49.2
F2Ag	4.258	63.3	0.549 (12.9%)	248.8	1.205 (28.3%)	335.8	0.387 (9.1%)	2.117	50.3
F3	3.570	58.7	0.537 (15.1%)	253.8 270.5	0.493 (13.8%) 0.505 (14.2%)	346.0	0.482 (13.5%)	1.553	56.5
F3Ag	4.392	80.3	0.540 (12.3%)	-	-	282.0	1.725 (39.3%)	2.127	51.6

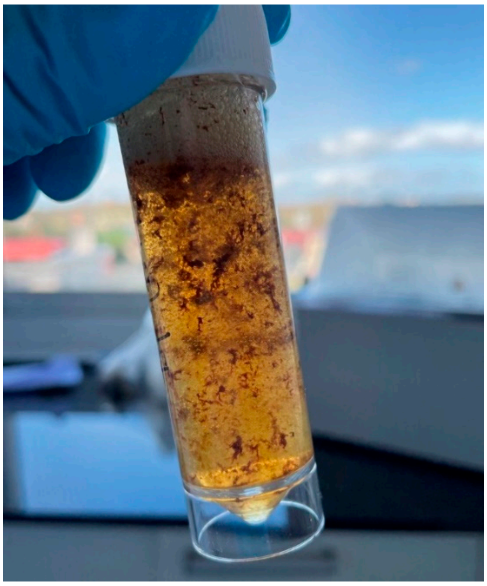

Figure S1. Compatibility tests of hybrid nanocomposites and culture medium, tryptic soy broth (TSB).

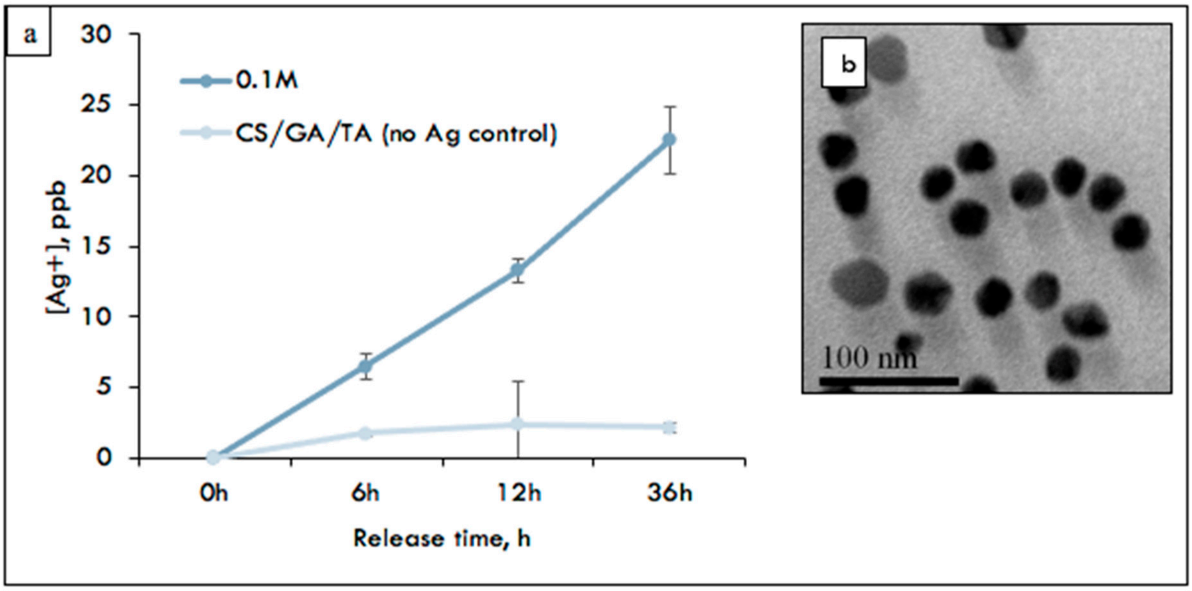

Figure S2. (a) Silver ion release from CS/GA/TA/AgNPs (dark blue), control CS/GA/TA (light blue) and (b) TEM micrograph of the core-shell structure of CS/GA/TA/AgNPs after the synthesis.

The ion release from several selected nanocomposites composed of nanosilver core and several organic shells was assessed by Electrothermal Atomic Absorption Spectroscopy (ETAAS). The sample of thin film with 60 mm diameter was completely immersed in 10 mL of contact solution, which is a 1:1 mixture of phosphate buffer (pH 6.8, ionic strength 0.1) and 0.85% w/w NaCl. The samples in test tubes were shaken manually every 2 h and right before sampling. The sampling took place at 6 h, 12 h, 36 h. The control sample without addition of silver precursor and presence of AgNPs was used, named CS/GA/TA. The experiments were carried out in triplicates.
